# Adsorption of Amorphous Silica Nanoparticles onto Hydroxyapatite Surfaces Differentially Alters Surfaces Properties and Adhesion of Human Osteoblast Cells

**DOI:** 10.1371/journal.pone.0144780

**Published:** 2016-02-10

**Authors:** Priya Kalia, Roger A. Brooks, Stephen D. Kinrade, David J. Morgan, Andrew P. Brown, Neil Rushton, Ravin Jugdaohsingh

**Affiliations:** 1 Division of Trauma & Orthopaedic Surgery, University of Cambridge, Cambridge, United Kingdom; 2 Department of Chemistry, Lakehead University, Thunder Bay, Ontario, Canada; 3 Cardiff Catalysis Institute, School of Chemistry, Cardiff University, Cardiff, United Kingdom; 4 School of Chemical and Process Engineering, University of Leeds, Leeds, United Kingdom; 5 MRC Human Nutrition Research, Elsie Widdowson Laboratory, Cambridge, Cambridge, United Kingdom; University of Oulu, FINLAND

## Abstract

Silicon (Si) is suggested to be an important/essential nutrient for bone and connective tissue health. Silicon-substituted hydroxyapatite (Si-HA) has silicate ions incorporated into its lattice structure and was developed to improve attachment to bone and increase new bone formation. Here we investigated the direct *adsorption* of silicate species onto an HA coated surface as a cost effective method of incorporating silicon on to HA surfaces for improved implant osseointegration, and determined changes in surface characteristics and osteoblast cell adhesion. Plasma-sprayed HA-coated stainless steel discs were incubated in silica dispersions of different concentrations (0–42 mM Si), at neutral pH for 12 h. Adsorbed Si was confirmed by XPS analysis and quantified by ICP-OES analysis following release from the HA surface. Changes in surface characteristics were determined by AFM and measurement of surface wettability. Osteoblast cell adhesion was determined by vinculin plaque staining. Maximum Si adsorption to the HA coated disc occurred after incubation in the 6 mM silica dispersion and decreased progressively with higher silica concentrations, while no adsorption was observed with dispersions below 6 mM Si. Comparison of the Si dispersions that produced the highest and lowest Si adsorption to the HA surface, by TEM-based analysis, revealed an abundance of small amorphous nanosilica species (NSP) of ~1.5 nm in diameter in the 6 mM Si dispersion, with much fewer and larger NSP in the 42 mM Si dispersions. ^29^Si-NMR confirmed that the NSPs in the 6 mM silica dispersion were polymeric and similar in composition to the larger NSPs in the 42 mM Si dispersion, suggesting that the latter were aggregates of the former. Amorphous NSP adsorbed from the 6 mM dispersion on to a HA-coated disc surface increased the surface’s water contact angle by 53°, whereas that adsorbed from the 42 mM dispersion decreased the contact angle by 18°, indicating increased and decreased hydrophobicity, respectively. AFM showed an increase in surface roughness of the 6 mM Si treated surface, which correlated well with an increase in number of vinculin plaques. These findings suggest that NSP of the right size (relative to charge) adsorb readily to the HA surface, changing the surface characteristics and, thus, improving osteoblast cell adhesion. This treatment provides a simple way to modify plasma-coated HA surfaces that may enable improved osseointegration of bone implants.

## Introduction

Silicon is reported to be an important micronutrient for bone and connective tissue health [1–5]. In bone, Si is present at the growth front, where it is involved in early stages of bone calcification/mineralisation [1]. *In vitro* studies have also demonstrated a (direct) stimulatory effect of Si on bone-forming osteoblasts [6–8]. The beneficial effect of silicon on bone and on osteoblast cells led to the development of Si-substituted calcium phosphates as an improved bone substitute material [9–14], with the first, phase-pure, Si-substituted hydroxyapatite (Si-HA) reported by Gibson *et al*. in 1999. Indeed, *in vivo* studies have shown Si-HA to significantly increase new bone formation, compared to unsubstituted hydroxyapatite (HA) [15, 16], while *in vitro* studies have reported stimulatory effects of Si-HA on bone forming and bone resorbing cells [16, 17]. Currently, porous Si-HA granules are available commercially for clinical applications (Actifuse™, Baxter, UK). The application of Si-HA has also been extended to metal implants with the demonstration of Si-HA plasma-sprayed coatings [9].

Silicon substituted HA materials have silicate ions incorporated into the lattice structure [18], and it is suggested that these materials function as a long-term Si delivery system, but exactly how dissolved silica interacts with HA and cells to produce its documented osteogenic effect is still poorly understood. There is evidence that the dissolved silica is re-adsorbed on to the Si-HA surface [10] and may act as a nucleation site for new bone formation/bone attachment, and some research groups are currently investigating methods of depositing layers of silica onto implant surfaces for enhanced bone attachment [11, 12]. We wished to investigate the adsorption of silica species onto the surface of plasma-sprayed HA coating on a biocompatible metal and its effect on cells for two reasons: (1) to provide insight into how silicon may interact with cells after its release from SiHA and (2) to show that it could be a cost-effective method for modifying the surface of HA-coated implants to improve their osseointegration. Here we demonstrate the direct adsorption of silica on a plasma-sprayed HA coating, which may provide the benefits of silica without altering the lattice structure of HA. Changes to surface characteristics (wettability and topography), dissolution and cell adhesion were investigated. This study improves our knowledge of Si-calcium phosphate (bone) interactions with HA and may provide an economical method of incorporating silicon to HA-coated surfaces.

## Materials and Methods

### Materials

Sodium silicate concentrate (7 M, pH 14), HCl and NaOH, were all obtained from Sigma-Aldrich UK. High purity (HP) water (18 MΩ cm^-2^) was obtained from a MilliQ water purification system (Millipore, UK). Polypropylene tubes (13 mL) were purchased from Sarstedt Ltd. (Basingstoke, UK). McCoy’s 5A media was from Bioconcept (Allschwil, CH), and fetal bovine serum (FBS) and penicillin/streptomycin/glutamine were from Invitrogen (Paisley, UK). Simulated body fluid (SBF) was prepared according to Kukubo *et al*. [13]. For cell isolation and culture, see S1 Methods.

### Aqueous silica dispersions

Silica dispersions (0–42 mM Si) were prepared at room temperature by dilution of the sodium silicate concentrate in HP water and then immediately neutralising to pH 7.3 using HCl and NaOH (tested for low Si content). The dispersions were used within 12 h of preparation. To enable ^29^Si NMR analysis (see below), 6 and 42 mM dispersions were similarly prepared from a silicate concentrate which contained 1.5 M ^29^SiO_2_ (99.96 atom% ^29^Si; Isonics) and 1.5 M KOH (99.99%, Sigma Aldrich).

### Nuclear magnetic resonance spectroscopy (^29^Si-NMR)

Nuclear magnetic resonance spectroscopy of ^29^Si (^29^Si-NMR was used to analyse 6 and 42 mM ^29^Si-enriched silica dispersions. 6 h after each dispersion had been prepared, it was loaded into a Si-free 10 mm O.D. Kel-F NMR tube and analysed (99.35 MHz) at 25°C on a Bruker AMX500 spectrometer with a Si-free probe head, employing 5930 π/2 observe pulses and a 53 s inter-pulse period (total acquisition = 87 h). ‘Snapshot’ spectra were recorded at the beginning, middle and end of the longer acquisition to ensure that the species distribution of the dispersion had not significantly changed.

### Molybdic acid assay

The dissolved Si fraction (“monomeric silica”) in the dispersions was assayed as described elsewhere [19, 20], relying on the formation of a yellow coloured silicomolybdate complex that can be detected spectrophotometrically at 410 nm.

### Ultra-filtration

Silica dispersions containing 0, 6 or 42 mM Si were freshly prepared in triplicate. After 12 h, aliquots were loaded into Vivaspin-6 centrifugal concentrators (Sartorius Stedim Biotech) containing 10, 50, 100 or 1000 kDa MWCO polyethersulfone ultrafilter membranes, which have nominal pore sizes (measured by EM) equal to 2.5, 4, 10 or 100 nm, respectively [14]. They were then centrifuged at 3,800 RPM at room temperature for 10 min and analysed for total Si concentration by ICP-OES (below).

### Transmission Electron Microscopy (TEM)

Transmission electron microscopy was employed to image particles formed in silica dispersions. Ultrafiltered solutions were drop-cast as suspensions onto holey carbon support films on copper grids (Agar Scientific Ltd.) and allowed to air dry before imaging on a FEI CM200 field emission gun microscope. For elemental mapping of silica nanoparticle (NSP), energy-filtered TEM (EF-TEM) Si *L*_2,3_ images were recorded with an 8.4 mrad objective aperture and a 10 eV slit centred at 82, 92 and 119 eV energy loss. Measurement of particle sizes in both ultrafiltered 6 mM and 42 mM silica dispersions (n = 13) was carried out using ImageJ (National Institutes of Health, USA).

### Stainless steel (316L) discs

Stainless steel discs 1 mm in thickness and 10 mm in diameter were fabricated by Precision Medical Engineering (Cambridge, UK) and HA coatings were plasma-sprayed with HA by Plasma Biotal (Buxton, UK). Uncoated discs were prepared by removing the HA with 0.5 M HCl. Discs were washed/sterilised in an ultrasonic bath with successive treatments of HP water, 70% industrial methylated spirit (IMS) and 100% IMS.

### X-ray diffraction (XRD) of plasma-sprayed HA coatings

The crystallinity of the HA coatings was measured relative to the unsintered powder used for spraying. After plasma-spraying, the coating was removed from the substrate, ground to less than 38 μm particle size (as determined by sieving), and then packed 10 mm x 20 mm x 1 mm thick into a powder sample holder for x-ray diffraction analysis with a PANalytical X-ray diffractometer (Cambridge, UK) and were compared to Joint Committee on Powder Diffraction Standard HA (009–0432), tricalcium phosphate (009–0169) and calcium oxide phosphate (025–1137).

### Silica-treated stainless steel discs

Silica-treated stainless steel discs (HA-coated and uncoated) were individually prepared by soaking in 3 mL of a freshly prepared silica dispersion for 12 h at 25°C in 13 mL polypropylene tubes, and were then dipped in three changes of HP water to remove non-adsorbed Si. The amount of silica adsorbed on a disc was determined by soaking it in 3 mL 0.5 M NaOH for 24 h at 25°C and using ICP-OES (below) to analyse the resulting solution.

### Inductively coupled plasma optical emission spectroscopy (ICP-OES)

Inductively coupled plasma optical emission spectroscopy (ICP-OES) was used to determine the total Si content of aqueous silica dispersions and other solutions, following dilution 1:1 with 2.5% nitric acid. Analysis was carried out at 251.611 nm on a Jobin Yvon JY2000-2 ICP-OES spectrophotometer using 3 mL sample portions, 1 mL/min flow rate, 0.08 nM peak profile window, 21 increments per profile, 0.5 s integration per increment, and appropriately prepared matrix-matched standards.

### X-ray photoelectron spectroscopy (XPS)

Surface concentrations of Si, calcium (Ca) and phosphorus (P) on silica treated discs were measured by X-ray photoelectron microscopy (XPS) using a Kratos Axis Ultra-DLD XPS spectrometer with 1486.6 eV monochromatic Al Kα radiation and 40 eV pass energy. Charge correction was performed using the Kratos 'snorkel' lens system. Spectra were referenced to the C(1s) line at 284.7 eV, and processed with CasaXPS software (v. 2.3.15, using sensitivity factors supplied by the manufacturer) after correction for transmission function, angle and escape-depth dependence.

### Water contact angle

Water contact angle was measured for HA-coated discs using a KSV Instruments KSVCAM 200 optical contact angle camera (v. 4.0 software) and 0.1 mL water drop size.

### Atomic force microscopy (AFM)

Atomic force microscopy (AFM) of HA-coated discs was carried out using a Veeco Multimode AFM with a contact mode alumina tip. The treated surfaces were both hard and consolidated enough to withstand the force exerted at the tip. Surface roughness was measured using a silicon nitride tip. All AFM image and surface roughness analysis was performed using WSxM software [21].

### Silica release in simulated body fluid

Silica treated HA-coated discs were washed, placed in 24-well culture plates, air-dried in a laminar flow hood for 24 h and then incubated for periods ranging from 1 min to 168 h under standard cell culture conditions (37°C, 5% CO_2_) in either (a) simulated body fluid (SBF) at pH 7.25, (b) McCoy’s 5A medium supplemented with 10% FBS, 30 μg/mL L-ascorbic acid 2-phosphate and 1% penicillin/streptomycin/glutamine, or (c) the same cell culture medium described in (b), but also with primary human osteoblasts (HOB) cultured on the surface (10^4^ cells/disc, primary osteoblast isolation and culture are described in **S1 Methods**). SBF has an ion content similar to that of human blood plasma [13], and was used here to mimic the immediate environment around an implant post-implantation. Thereafter, the discs were removed and the solutions analysed for total Si content by ICP-OES.

### Vinculin plaque analysis

Human osteoblast (HOB) cells were seeded onto silica treated HA-coated discs (0, 6 or 42 mM Si; n = 3) at a density of 10^4^ cells / disc / well in 24-well TCP plates, and cultured in McCoy’s medium, as above for 24 h or 48 h. Human osteoblasts were then stained for vinculin, a protein associated with focal adhesion complexes [22], and were detected and visualised for quantification by immunofluorescent staining. Briefly, cells were fixed in 4% fresh paraformaldehyde, treated twice with permeabilisation buffer with a 10.3% sucrose solution containing 0.3% NaCl, 0.6% MgCl_2_, 4.8% HEPES and 0.5% Triton-X at 4°C for 5 min, and blocked with 1% bovine serum albumin (BSA)-PBS for 10 min at room temperature. Cells were then treated with primary FITC-conjugated vinculin antibody (1:50 dilution 1% w/v BSA in PBS) for 2 h at room temperature and washed three times with 0.05% Tween-20 in PBS. Cells were visualised using a Leica DM RXA2 microscope and photographed in a 5 x 4 grid using a Q Imaging Retiga EXi camera and Surveyor Workspace Viewer (version 5.5.5.37, Objective Imaging) at 20x magnification. Four discs were used per silica treatment and the number of vinculin plaques in each cell was counted in six randomly selected squares from the grid chosen using a web-based algorithm[23]. Plaques were characterised in accordance with their morphology [19] and location (nuclear, peripheral or cytoplasmic). The mean number of vinculin plaques per cell, for each silica treatment, was used for statistical analysis. In addition the mean number of cells on each surface over time (4 h to 14 days) was quantified using the CyQuant cell proliferation assay (see **S1 Methods**).

### Statistical analyses

Statistical analyses were performed in SPSS 17.0 for MS Windows. The Kruskal-Wallis test was performed to compare more than two groups, with *P* ≤ 0.05 being considered significant. *Post-hoc* tests were then performed if differences were found (*P* ≤ 0.05) using the Mann Whitney U test.

## Results and Discussion

### Plasma sprayed HA coating

**Fig 1:**
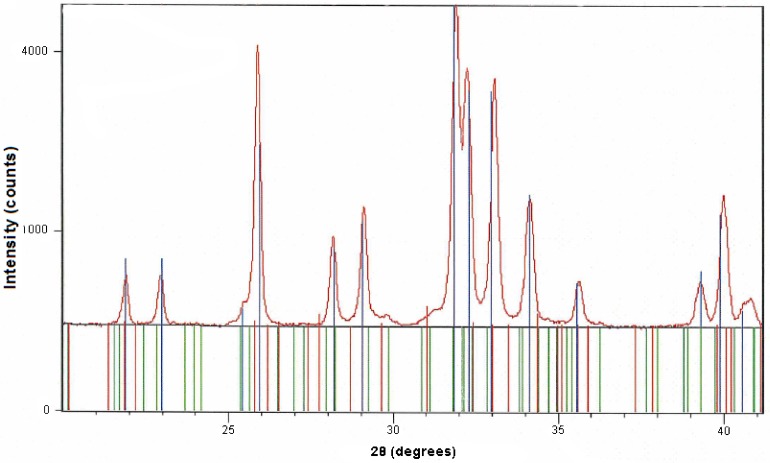
XRD analysis showing phase purity of HA coating after plasma-spraying on to stainless steel discs. In this sample, crystallinity of the HA was 99.6% following plasma-spraying. The brown line shows the experimental XRD scan of the sample and the vertical blue lines indicate the match with Joint Committee on Powder Diffraction Standards reference HA. Standard tricalcium phosphate (red) and calcium oxide phosphate (green) are also shown.

XRD analysis of the plasma-sprayed HA coating suggested a limited glassy phase (**Fig 1**), slightly lower than the primary powders that were used/plasma sprayed (data not shown). This indicates that the process of plasma spraying, which utilises high temperature and rapid cooling, resulted in the loss of HA crystallinity previously been reported by others [9, 24].

### Characterisation of silica dispersions

**Fig 2:**
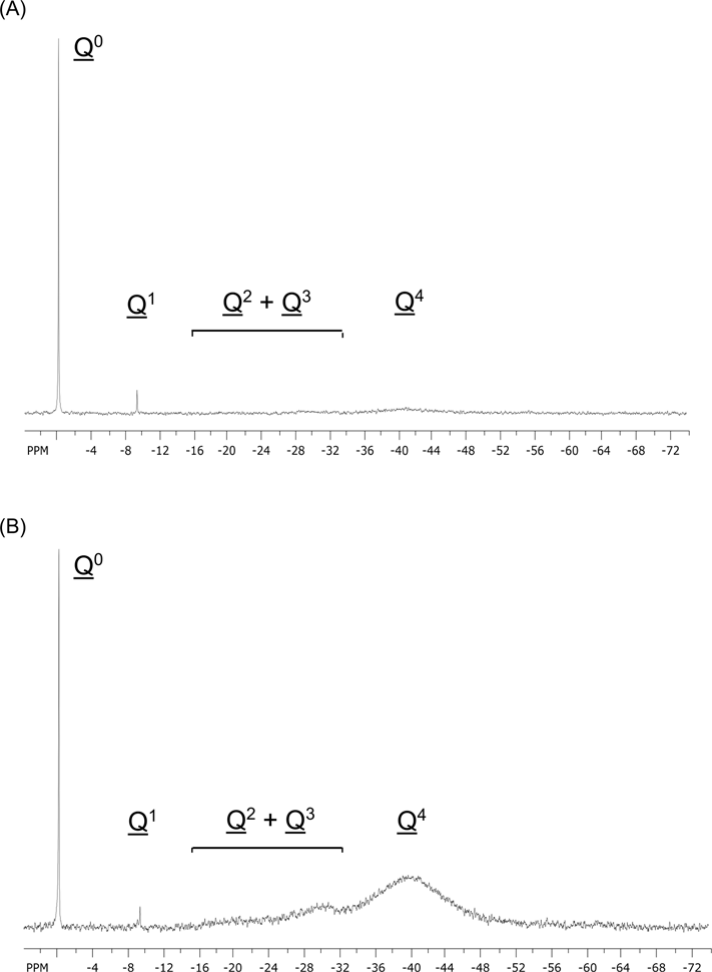
^29^Si NMR spectra (99.35 MHz) at 25°C of a) 6 and b) 42 mM silica dispersions. Q^*n*^ represents a Si atom with *n* coordinated -OSi groups.

**Fig 3:**
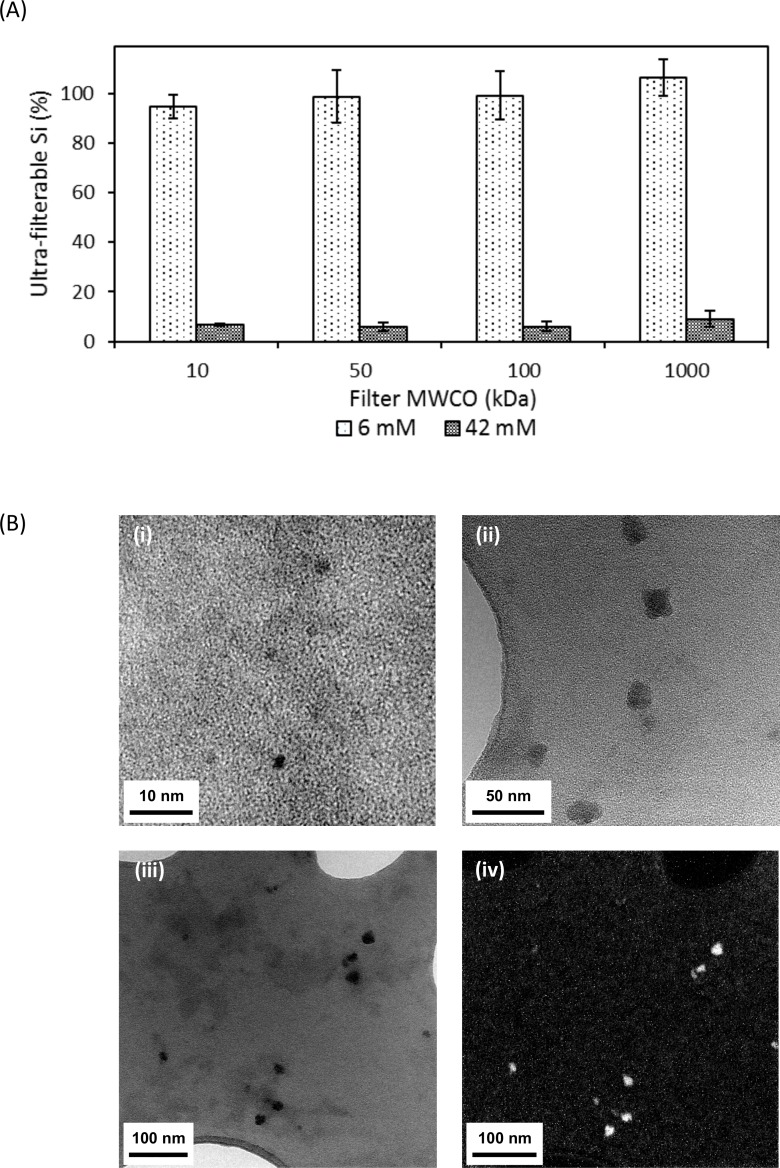
(A) Percentage of total silica in 6 and 42 mM silica dispersions that passed through 10, 50 100 and 1000 kDa MWCO (molecular weight cut-off) membranes. (B) TEM analysis of the filtrates. i) TEM of amorphous nanoscale silica particles (NSP) in the filtrate of the 10 kDa MWCO filtered 6 mM silica dispersion; ii) TEM showing larger (< 20 nm) NSP in the filtrate of the filtered 42 mM silica dispersion; iii) Bright field, zero loss filtered TEM image of NSP in the filtrate of the filtered 42 mM dispersion and iv) EF-TEM Si *L*_2,3_ elemental map image of NSP shown in (iii). The bright regions indicate Si-rich areas.

Silica dispersions which had been freshly prepared to contain up to 42 mM Si were completely transparent and showed no signs of precipitation. ^29^Si-NMR spectroscopy revealed that they were each saturated in orthosilicic acid (H_4_SiO_4_) and disilicic acid (H_6_Si_2_O_7_), accounting for approximately 3 mM of the total Si content (**Fig 2**). The remainder existed as nanoscale silica particles (NSP; **Table 1)**. The NSP in each dispersion exhibited a similar extent of polymerization, containing 82% fully-condensed SiO_4_ tetrahedra (Q^4^) and 18% partially-condensed tetrahedra (Q^2^ and Q^3^). However, their average particle size increased with the Si concentration of the dispersion. Since roughly 96% of the total Si content in a 6 mM dispersion could pass through even a 10 kDa MWCO filter membrane (**Fig 3A**), 92% of the solid silica occurred as NSP with diameters < 2.5 nm. This was confirmed by TEM image-based analysis of particle size in the filtrate of the 6 mM dispersion, which revealed monodisperse spherical particles that were ~ 1.5 nm (1.48 ± 0.22 nm) in diameter (**Fig 3B**). By contrast, few NSP (< 10%) in the 42 mM dispersion could pass through a 1000 kDa MWCO filter membrane, suggesting that the majority of the NSP were on the order of 100 nm or larger in diameter. Again, TEM image-based analysis of the filtrate revealed (i) low levels of NSP and (ii) NSP of ~ 20 nm (19.5 ± 3.61 nm) in diameter (**Fig 3B**), although it is not known how much the drying of the specimen for TEM might have affected the particle size. EF-TEM confirmed that these larger particles were Si-rich (**Fig 3B**). The fact that particle size, but not extent of polymerization, increased with Si concentration would suggest that the particles grow through aggregation of the primary *ca*. 1.5 nm particles detected in the 6 mM dispersion. This conclusion is supported by the observation that variously concentrated silica dispersions exhibited nearly identical rates of particle dissolution at pH 7.2 following dilution to 1 mM, *i*.*e*., to below the silica saturation level (**S1 Fig**).

**Table 1:** Distribution of silicon content in freshly prepared silica dispersions.^a^

Silica dispersion(mM Si)	Si distribution (%)			NSP composition ^b^ (%)	
	H_4_SiO_4_	H_6_Si_2_O_7_	NSP	Q^2^ + Q^3^	Q^4^
6	47.0 ± 2.5	2.3 ± 1.7	50.7 ± 2.5	17 ± 3	83 ± 2
42	5.7 ± 0.3	0.38 ± 0.03	93.7 ± 1.4	18 ± 2	82 ± 1

^***a***^ Determined by ^29^Si NMR spectroscopy.

^***b***^ Q^*n*^ represents a Si atom with *n* coordinated -OSi groups. NSP = nanoscale silica particles

### Adsorption of silica dispersion to HA-coated surface and changes in surface characteristics

**Fig 4:**
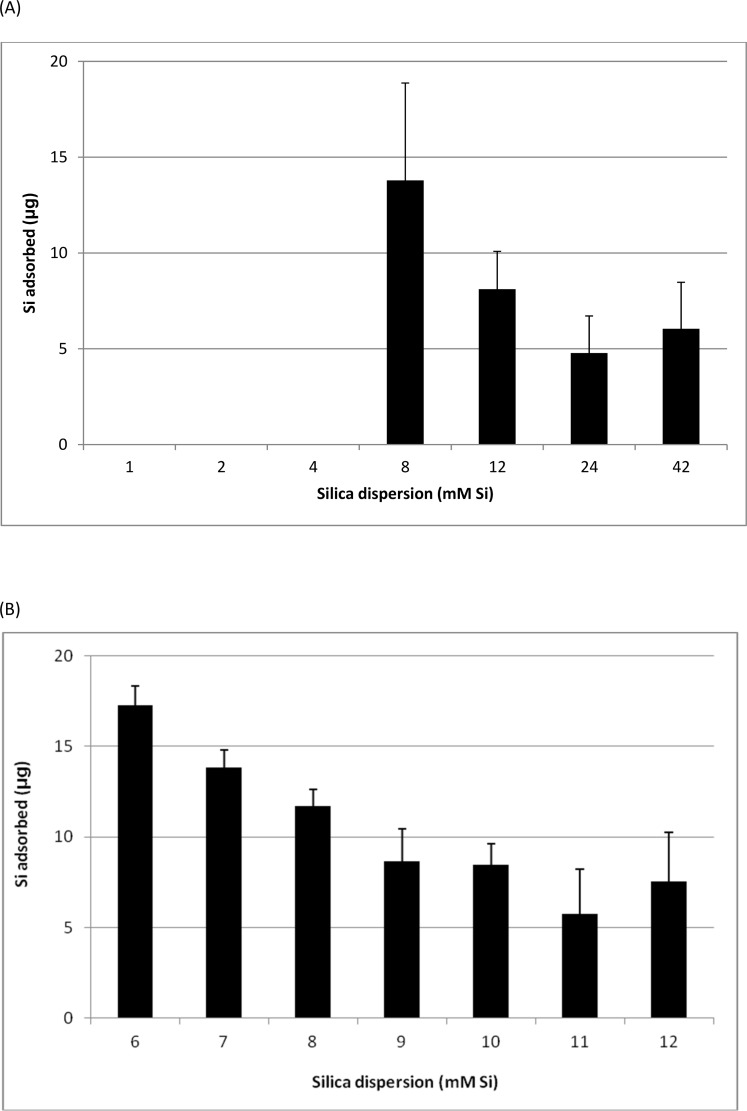
Silicon adsorbed onto HA-coated stainless steel discs following incubation in silica dispersions containing (A) 1 to 42 mM Si and (B) 6 to 12 mM Si. Data were corrected for adsorbed Si from the control (0 mM Si) solution. Data are means ± SD of *n* = 4 discs per treatment.

The amount of silica that was adsorbed onto stainless steel discs was determined by soaking the treated discs in 5 M sodium hydroxide, and the Si released quantified by ICP-OES. In the case of uncoated discs, negligible adsorption of Si was detected from any of the silica dispersions (data not shown). HA-coated discs only took up Si from silica dispersions containing > 4 mM Si (**Fig 4A**). HA-coated discs exhibited the greatest level of adsorption when treated with a 6 mM silica dispersion, despite this being the least concentrated of all the NSP-containing treatments that were tested (**Fig 4B**). As described above, this treatment contained the smallest (*ca*. 1.5 nm) silica particles. As the silica concentration of the treatments was increased up to 10 mM, the extent of adsorption decreased monotonically to about half that observed for the 6 mM treatment. Further increases in silica concentration (up to 42 mM) yielded no additional adsorption (**Fig 4A**). XPS measurements of the silica treated HA-coated discs confirmed that the 6 mM treatment yielded the highest amount of silica adsorption (**Table 2**). Although a range of silica concentrations were studied initially, the concentrations which showed maximal and minimal adsorption to the HA surface was selected for further investigation (6 mM and 42 mM Si).

**Table 2:** Surface characteristics of HA-coated stainless steel discs following nanoscale silica particle (NSP) treatment.

Silica dispersion(mM Si)	Surface atomic ratio^a^Si: Ca: P	Water contact angle^b^/deg	Surface roughness ^c^ (nm)
0	0.31: 1.5: 1	37 ± 9	9.7 ± 0.5
6	11.3: 1.7: 1	90 ± 6	44 ± 1
42	1.9: 1.5: 1	19 ± 4	22 ± 2

^*a*^ By XPS, in the first 10 nm; *n* = 2 discs per treatment.

^*b*^ *n* = 3 discs per treatment.

^*c*^ By AFM; *n* = 4 images per treatment.

**Fig 5:**
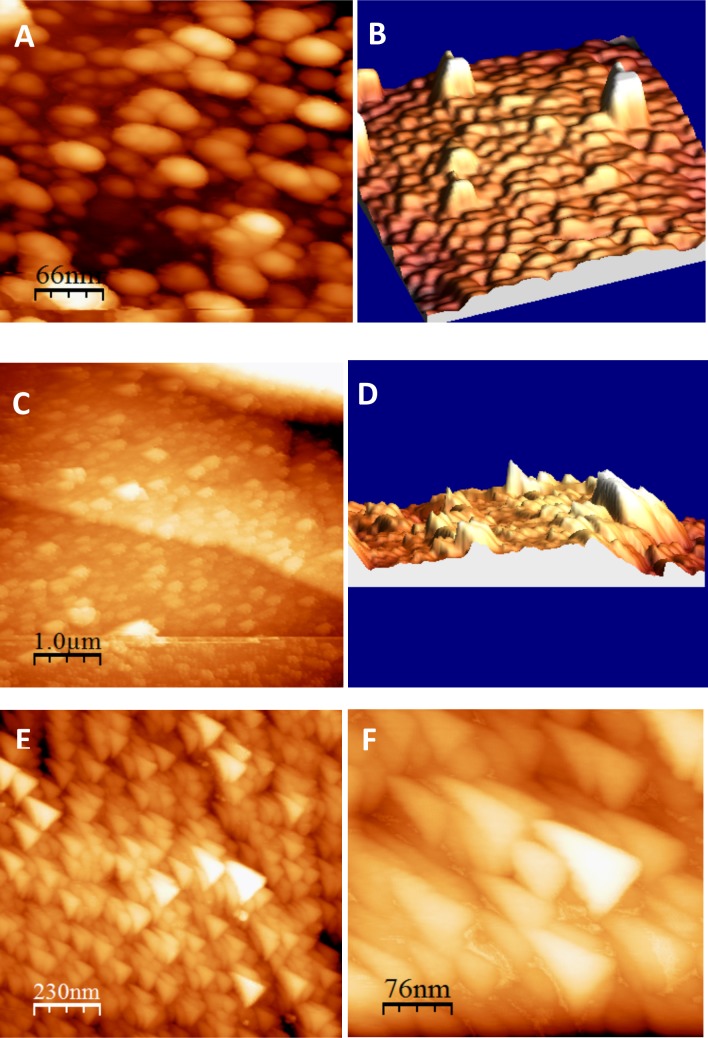
Representative images of atomic force microscopy (AFM) carried on out plasma-sprayed HA surfaces following incubation in 0 (A, B), 6 (C, D) and 42 mM (E, F) Si dispersions for 12 h and air-dried. Actual roughness measurements are presented in **Table 2**.

Water contact angles were measured to determine differences between the surface wettability of HA-coated discs treated with silica dispersions containing 0, 6 or 42 mM Si (**Table 2**). The 42 mM silica treatment yielded a lower water contact angle than the 0 mM control treatment (19 and 37°, respectively), implying that the treated surface was slightly more hydrophilic. By contrast, and surprisingly, treatment with the 6 mM silica dispersion yielded a higher contact angle (90°), suggesting a substantial decrease in hydrophilicity. AFM analysis of the 6 mM silica treated HA surface suggested an increase in surface roughness (**Fig 5C** and **5D**) compared to the untreated surface (**Fig 5A** and **5B**, **Table 2**).The 42 mM silica treatment also yielded an increase in surface roughness, but less than the 6 mM silica treatment (**Fig 5E and 5F**).

### Dissolution of the silica adsorbed from the HA coated surface

**Fig 6:**
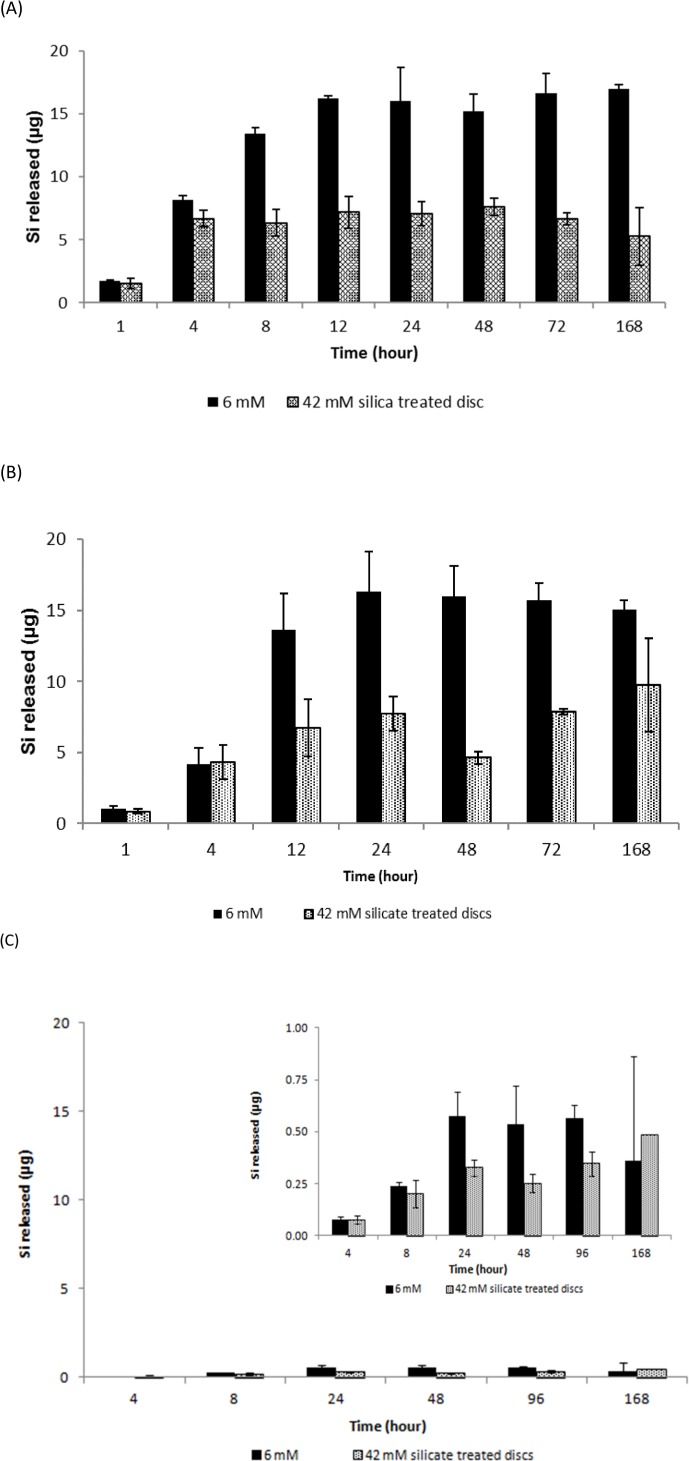
Release of Si from silica-treated HA-coated stainless steel discs as a function of time: A) into SBF at pH 7.2 and 25°C; B) into McCoy’s 5A supplemented medium at pH 7.4, 37°C and 5% CO_2_; and C) in McCoy’s 5A medium at pH 7.4, 37°C and 5% CO_2_ in the presence of primary human osteoblasts (inset shows an increased y-scale). Data are means ± SD of *n* = 3 discs per time point, and were corrected for adsorbed Si from the control (0 mM Si) solution.

Next, the release (or solubility) of the adsorbed silica, from the silica-treated HA surfaces was determined under circum-neutral pH and at 37°C in SBF (**Fig 6A**) and in primary human osteoblast cell culture medium (**Fig 6B**). Comparison with **Fig 4** revealed that silica adsorbed from the 6 mM dispersion was completely released in SBF by 12 h and in the osteoblast culture medium by 24 h. Similarly, the comparatively small amount of silica that was adsorbed from the 42 mM treatment was completely released between 4 and 8 h. Interestingly, when primary human osteoblast cells were cultured on the silica-treated surfaces the proportion of Si released into cell culture media, or potentially absorbed by cells, was drastically reduced (**Fig 6C**).

### Cell adhesion on the silica adsorbed HA coated surface

**Fig 7:**
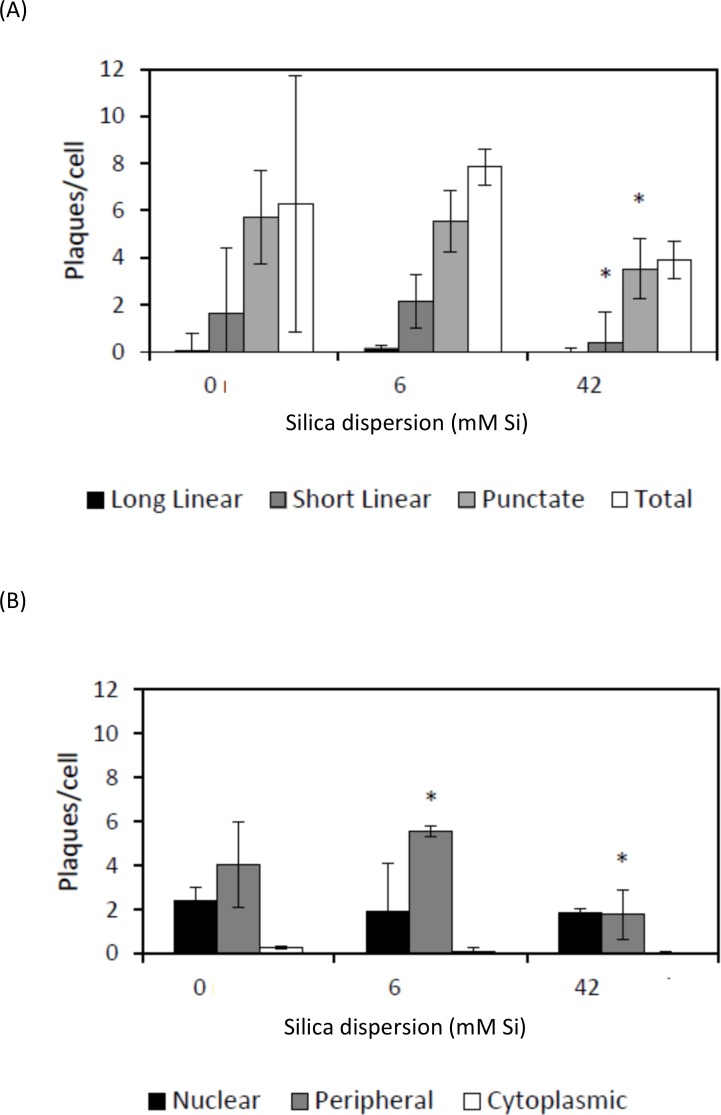
Number of vinculin plaques per human osteoblast cell after 48 h growth on HA-coated discs treated with 0, 6 or 42 mM silica dispersion. Plaques were sorted A) by shape (long, short or punctuate) and B) by cell location (peripheral, nuclear or cytoplasmic). Significant differences (*P* ≤ 0.05) between silica treatment and control (0 mM Si) are represented by an asterisk.

Although at 24 h there were no significant differences in the number or distribution of vinculin plaques between treatments (data not shown), at 48 h the differences were pronounced (**Fig 7)**. Examples of the different types of vinculin plaque morphologies are illustrated in **S2 Fig** The 42 mM Si treatment yielded significantly fewer plaques compared to the control treatment (*P* <0.05; **Fig 7A**) and this was mainly due to a significant drop in the number of peripherally located plaques (*P* < 0.05; **Fig 7B**). In contrast, with the 6 mM Si treatment, there was no significant difference in the total number of plaques or distribution in plaque shape. However, there was a change in the distribution of plaque location, with significantly more peripherally located plaques compared to the control treatment (*P* < 0.05; **Fig 7B**). A preliminary cell proliferation study using the CyQuant assay showed an increase in proliferation on the silica-treated HA-surfaces with time until 7 days after cell seeding (**S3 Fig**).

In summary, we have demonstrated the adsorption of amorphous silica nanoparticles onto plasma-sprayed HA-coated stainless steel surfaces. The lack of adsorption of silica species below 4 mM Si would suggest that negatively charged polymeric (colloidal) silica species have higher affinity for the HA surface than neutral dissolved species at ca. pH 7 (Table 1 and Fig 2). The divergent results obtained for the 6 and 42 mM silica treatments would further suggest that smaller NSP may have a more optimal size to charge ratio for the plasma-sprayed HA surface. Adsorption of NSP altered the wettability of the HA surface, as well as surface roughness and cell adhesion. Indeed, cells grown on the HA surfaces appear to prefer the rougher and less hydrophilic surface of the 6 mM silica treated HA surface. Generally, cells prefer hydrophilic surfaces [20, 25]. Interestingly, culture of osteoblast cells onto the Si-adsorbed surfaces reduced the dissolution of Si from the surface into the cell culture media. Whether this is due to osteoblast attachment preventing silica release from HA surfaces, or attributable to the uptake of silica into cells is not known and should be investigated in future studies by measuring the Si content of cells. However, the increase in osteoblast cell adhesion on the 6 mM silica treated HA surface would suggest that it has a more favourable surface than HA alone and may benefit osteoblast cell growth and new bone formation.

## Supporting Information

S1 Fig Increase in the monomeric silica content (by molybdic acid assay) of several different silica dispersions following dilution at pH 7.2 to 1 mM Si. (DOCX)

S2 Fig Examples of the different types of vinculin plaque morphologies. A) After 24 hours’ culture on glass coverslips, short linear plaques are indicated with white arrows and long linear plaques with white asterisks (*). B) After 48 hours’ culture on HA surface (0 mM Si), short linear plaques are indicated with white arrows and punctate plaques with white asterisks (*).
(DOCX)

S3 Fig Cell proliferation as measured by the CyQuant assay of primary human osteoblasts cultured on silica-treated HA-coated discs. h = hour, d = day.
(DOCX)

S1 Methods Description of methods for primary human osteoblast (HOB) isolation and culture, and the CyQuant cell proliferation assay. (DOCX)
