# Supplementary figures and images for: Adsorption of Amorphous Silica Nanoparticles onto Hydroxyapatite Surfaces Differentially Alters Surfaces Properties and Adhesion of Human Osteoblast Cells
Source: PLoS One. 2016 Feb 10;11(2):e0144780. doi: 10.1371/journal.pone.0144780 (PMC4749379; doi:10.1371/journal.pone.0144780)

S1 Fig


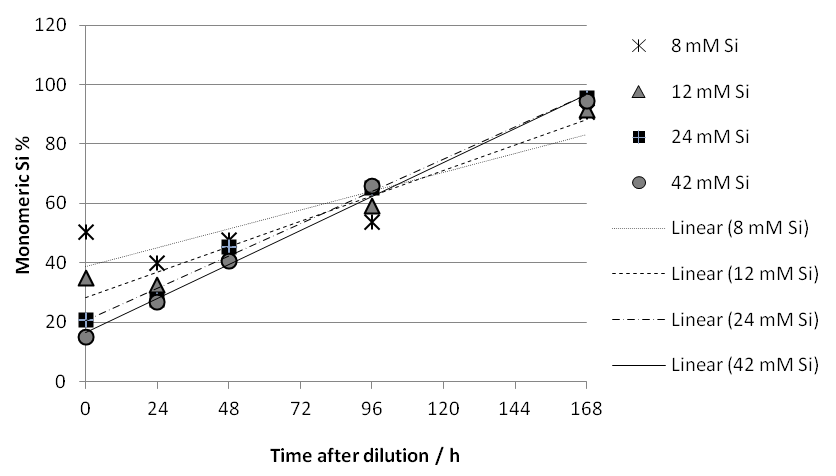

Supplement: S1 Fig — (DOCX) [file pone.0144780.s001.docx]

S2 Fig

**A**

**A**

200 μm


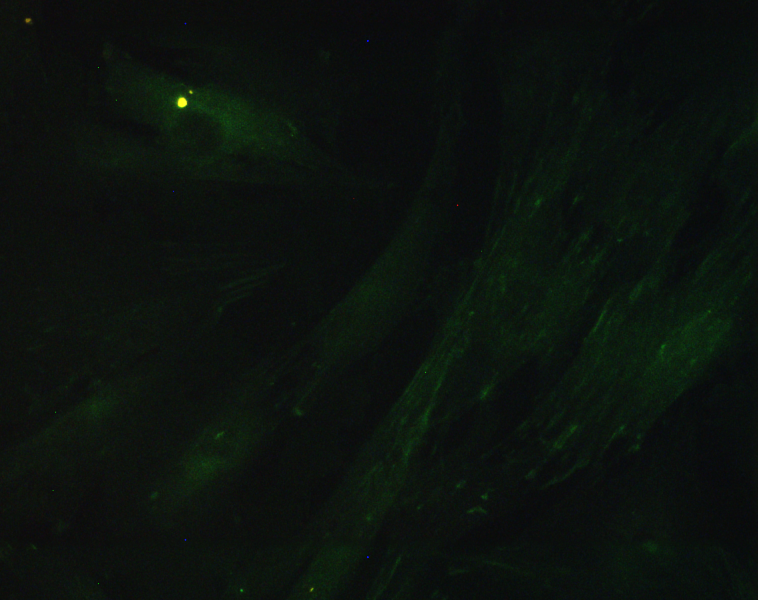


*****

*****


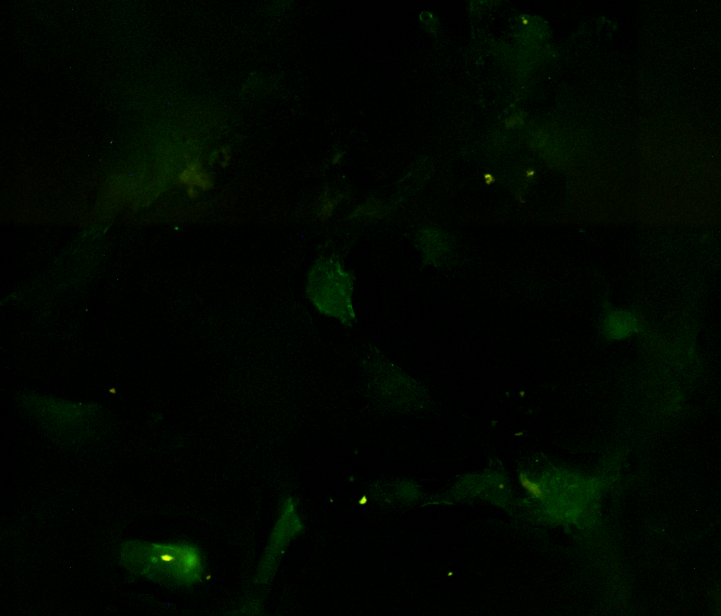


*****

*****

**B**

Supplement: S2 Fig — A) After 24 hours’ culture on glass coverslips, short linear plaques are indicated with white arrows and long linear plaques with white asterisks (*). B) After 48 hours’ culture on HA surface (0 mM Si), short linear plaques are indicated with white arrows and punctate plaques with white asterisks (*). (DOCX) [file pone.0144780.s002.docx]
