# Supplementary material for: Adsorption of Amorphous Silica Nanoparticles onto Hydroxyapatite Surfaces Differentially Alters Surfaces Properties and Adhesion of Human Osteoblast Cells
Source: PLoS One. 2016 Feb 10;11(2):e0144780. doi: 10.1371/journal.pone.0144780 (PMC4749379; doi:10.1371/journal.pone.0144780)
Supplement: S3 Fig — h = hour, d = day. (DOCX) [file pone.0144780.s003.docx]

S3 Fig
