# Supplementary material for: Adsorption of Amorphous Silica Nanoparticles onto Hydroxyapatite Surfaces Differentially Alters Surfaces Properties and Adhesion of Human Osteoblast Cells
Source: PLoS One. 2016 Feb 10;11(2):e0144780. doi: 10.1371/journal.pone.0144780 (PMC4749379; doi:10.1371/journal.pone.0144780)
Supplement: S1 Methods — (DOCX) [file pone.0144780.s004.docx]

# Supporting Information

## Supporting Methods

### Primary human osteoblast (HOB) cultures

HOB cells (passage 1) were obtained from discarded human femoral heads of consenting patients undergoing hip replacement surgery. The study was conducted according to the guidelines laid down in the Declaration of Helsinki and approved by the Cambridge University Hospitals NHS Foundation Trust and the Cambridge 2 Research Ethics Committee, UK (Ethics Number 06/Q0108/213). All participants gave signed written consent following oral and written explanation of the study details. Briefly, trabecular bone was removed from femoral head using a trephine and bone nibblers. Bone segments were rinsed in PBS, incubated with 1 mg/mL trypsin (Difco Trypsin 250, Becton Dickenson, Oxford, UK), rinsed in PBS, and then treated with 2 mg/mL dispase (Dispase II, Roche, Welwyn Garden City, UK). Next, they were washed in PBS, and then incubated with two successive aliquots of 2.8 mg/ml collagenase A (Roche). The two incubated collagenase solutions were pooled, centrifuged and the pellet resuspended in McCoy’s 5a medium (see **Materials and Methods**). Cells were seeded in 75cm^2^ flasks, grown until sub-confluent and detached using trypsin-EDTA (Invitrogen). Cells were then seeded (10^4^ cells/disc) on sterilised silica treated steel discs and cultured within individual wells of 24-well TCP plates, each containing 1 mL supplemented McCoy’s 5A medium (see **Materials and Methods**), and incubated under standard cell culture conditions (37 °C, 5% CO_2_).

## CyQuant Proliferation Assay

To specifically measure DNA content as a direct estimate of cell number, rather than DNA and RNA (where the levels of RNA may vary depending on many variables), RNase treatment was applied to the samples. This made the assay more specific to DNA content and so more closely representative of cell number. Briefly, after removal of medium following 4 h to 14 d incubation of cells on silica treated HA coated stainless steel discs, cells were washed with 1x PBS and then lysed in 1x CyQuant buffer (Invitrogen, Paisley, UK), and freeze-thawed at -80°C. CyQuant buffer was prepared from a 20x concentrated solution by dilution in DNA/RNase-free water. A 2x concentrated RNase incubation solution was prepared in 1x CyQuant buffer, with 360 mM NaCl, 2 mM EDTA, and 2.7 kilounits RNase. 25 µL freeze-thawed cell lysate per sample, in duplicate, was reacted with 25 µL 2x RNase incubation solution in a FluroNunc black plastic fluorometric 96-well plate. Samples were covered and incubated for 1 hour at room temperature, after which the RNase-treated lysates were reacted with 1+1 pre-prepared CyQuant dye. After further 2-5 min incubation at room temperature, the fluorescence was read in a fluorometer using 480 nm excitation and 520 nm emission wavelengths.
